# Nanostructured Zirconia Surfaces Regulate Human Gingival Fibroblasts Behavior Through Differential Modulation of Macrophage Polarization

**DOI:** 10.3389/fbioe.2020.611684

**Published:** 2021-01-20

**Authors:** Jincheng Wu, Pei Yu, Huling Lv, Shuang Yang, Zhe Wu

**Affiliations:** Guangzhou Key Laboratory of Basic and Applied Research of Oral Regenerative Medicine, Department of Prosthodontics, Affiliated Stomatology Hospital of Guangzhou Medical University, Guangzhou, China

**Keywords:** zirconia, macrophages, human gingival fibroblasts, cell adhesion, cell proliferation

## Abstract

Zirconia exhibits excellent biocompatibility and is widely used as dental implant materials in prosthodontics. Over the past years, research and development of dental implant biomaterials has focused on osseointegration, but few reports exist regarding the role of the immune environment on cellular responses to these materials. The present study investigates the effect of different nanostructured zirconia surface topographies on macrophage phenotypes and their influence on gingival fibroblast behavior. Three different nanostructured zirconia surfaces are characterized using scanning electron microscopy, atomic force microscopy, and water contact angle. Blank-machined zirconia (BMZ) surfaces were superior to RAW264.7 cell proliferation and adhesion. RAW264.7 seeded on all nanostructured zirconia surfaces polarized toward both inflammatory M1 and anti-inflammatory M2 macrophages with more M2 macrophage phenotype on BMZ surfaces. Meanwhile, conditioned media (CM) from RAW264.7 culture on three nanostructured zirconia surfaces inhibited cell apoptosis to human gingival fibroblasts (HGFs) but promoted HGF proliferation and secretion. Under modulation of RAW264.7 culture, HGFs cultured on BMZ surfaces significantly secreted more extracellular matrix with a higher expression of collagen-I (COL-I), vinculin (VCL), and fibronectin (FN) than those coated on self-glazed zirconia (CSGZ) and self-glazed zirconia (SGZ) surfaces. After being coated with a nano zirconia film, CSGZ surfaces showed certain changes in cell proliferation, adhesion, and protein production compared with SGZ surfaces. These findings will provide an overview of manipulating surface topography to modulate macrophage phenotypes in order to create an effective macrophage immune response and reinforce soft tissue integration.

## Introduction

Zirconia has gained outstanding popularity in recent years and is recommended as a dental material for implant because of its good biocompatibility (Zhang and Lawn, 2018), superior mechanical properties (Turon-Vinas and Anglada, 2018), low plaque affinity (Roehling et al., 2017), and excellent esthetic outcomes (Tabatabaian, 2019). Over the past years, research and development of dental implant biomaterials has focused on osseointegration. However, published studies addressing the effect of immune reaction on soft tissue integration are scarce. The biological seal in the transmucosal part of an implant acts as a biological barrier, prevents bacterial penetration, and protects the alveolar bone (Baltriukiene et al., 2014). The incidence of peri-implantitis was diagnosed in 31.2% of patients, and an undesirable clinical complication, such as soft tissue recession and marginal bone resorption, most often occurs as a result of inflammation, which threatens the long-term success of dental implants (Han et al., 2014; Bosshardt et al., 2017). Oftentimes, adverse immune reactions against foreign materials can lead to dramatic, immediate outcomes, such as intense pain, excessive inflammation, or rejection of the implanted material (Kzhyshkowska et al., 2015). Thus, sufficient soft tissue integration is essential to support the peri-implant tissues, improve esthetics, ensure a soft tissue seal against microorganisms, and preserve crestal bone level, ultimately increasing the longevity of the restoration (Atsuta et al., 2016).

A series of host responses can be elicited once surgical materials are implanted into living tissues (Anderson et al., 2008). Macrophages and fibroblasts are two regulatory cells participating in the host immune reaction to biomaterials (Glaros et al., 2009). Macrophages play a more prominent role in the immune responses, whereas fibroblasts are more significant during tissue remodeling (Witherel et al., 2019).

When the implant is inserted, macrophages derived from monocytes will first infiltrate rapidly to the implant site and attack foreign objects by producing various cytokines to regulate the immune microenvironment around the implant site (Zhou et al., 2015). Macrophages show phenotypic plasticity based on the topological structure of foreign materials and are classified into pro-inflammatory M1 and anti-inflammatory M2 types, with a vital role in disease, tissue healing following surgical injury, and biomaterial performance (Mosser and Edwards, 2008; Brown et al., 2012; Murray et al., 2014). M1 macrophages support inflammation by releasing pro-inflammatory cytokines, such as tumor necrosis factor-α (TNF-α) and inducible nitric oxide synthase (iNOS), while M2 macrophages promote tissue repair through producing tissue remodeling cytokines including IL-10, CD206, and transforming growth factor-β (TGF-β) that mediate cell migration, proliferation, and matrix remodeling (Galli et al., 2011; Wynn et al., 2013). Researches have claimed that shifting the macrophage phenotype from M1 to M2 was conducive to implant integration (Ma et al., 2014). In contrast, the presence of a great proportion of M1 relative to M2 is highly related to implant failure (Rao et al., 2012). The cytokines and growth factors secreted by M2 macrophages can support the migration, adhesion, and differentiation of human gingival fibroblasts (Wynn and Vannella, 2016).

Following modulation of macrophage phenotype polarization, gingival fibroblasts migrate to the wound site for the production of a new matrix and act as the major cell type responsible for creating a functional seal from the outside mucosa (Wang et al., 2016). It is well-known that surface properties may change macrophage functions such as cell survival, cell adhesion, and cytokine secretion and further regulate the adhesion and function of progenitor cells (Tan et al., 2006; Kearns et al., 2013). Modification of the processing methods may lead to different surface properties, subsequently influencing cell behavior (Rohr et al., 2020). Nanostructured surfaces have been considered to affect cell function in a different way in contrast to microscale surfaces (Xu et al., 2018). Reports discovered that an oriented alignment of human gingival fibroblasts can be induced on nanonet structuration of titanium surfaces, with more deposition of collagen (Llopis-Grimalt et al., 2019). Another report revealed that titanium surfaces coated with nanoscale silver possess antimicrobial efficacy and human gingival fibroblast cytocompatibility (Kheur et al., 2017). In addition, it is reported that TiO_2_ with nanotubes at diameters of 30 nm was conductive to induce macrophages to the M2 phenotype, leading to higher osteogenesis and better osseointegration in an *in vivo* study (Wang et al., 2018). Currently, the widely used zirconia is a representative of a technique based on a dry-pressing method, which is made by milling the partially interbred blanks followed by cold incrustation pressing, while self-glazed zirconia is formed by local plastic deformation introduced during a precision wet-chemistry process (Liu et al., 2016). At present, knowledge of how the nanostructured zirconia surfaces influence immune response and their interplay with fibroblast behavior is currently limited.

Thus, the aim of this study is 2-fold: first, the effect of nanostructured zirconia surface topography was investigated on macrophage polarization toward either an M1 or M2 macrophage phenotype. Thereafter, conditioned media (CM) collected from RAW264.7 culture on these nanostructured zirconia surfaces was harvested and cultured with gingival fibroblasts on their respective surfaces. Herein, we aim to investigate the macrophage phenotypes on nanostructured surfaces and to determine which implant surface tends to more favorably generate an optimal microenvironment from host macrophages for soft tissue cell integration.

## Materials and Methods

### Preparation of Nanostructured Zirconia Discs

All discs used in the experiment were provided by ErRan (Hanzhou, China) with a 20-mm diameter and a thickness of 1 mm. These tested discs fit directly to the bottom of 24-well culture plates. All discs were divided into three groups for the experiments: blank-machined zirconia (BMZ), self-glazed zirconia (SGZ), and coated self-glazed zirconia (CSGZ). The tissue culture plate (TCP) is used as a control group. Briefly, the BMZ surfaces were fabricated by a technique depending on a dry-pressing method. The SGZ surfaces were formed by the precision additive 3D gel deposition approach. The CSGZ surfaces were formed by self-glazed zirconia coated with a nano zirconia film. Prior to use, all discs were cleaned ultrasonically in acetone, anhydrous ethanol, and distilled water sequentially.

### Surface Characterization

#### SEM Measurements

The surface nanostructure of the blank-machined zirconia, self-glazed zirconia, and coated zirconia surfaces was characterized by using a scanning electron microscope (SEM, Gemini Sigma 300/VP, Zeiss, Oberkochen, Germany). The samples were washed with distilled water and acetone in an ultrasonic bath and dried at room temperature. The SEM observation was carried out on the surfaces. Accelerating voltages of 1 and 2 kV were applied in order to reduce the charging up of the samples.

#### AFM Measurements

The surface topography of the samples was characterized in a 2-cm × 2-cm area by an atomic force microscope (AFM, Dimension Icon, Finland and Sweden) in DC-EFM mode with scan rate of 0.8 Hz. The surface roughness was also characterized by a white light interferometer. Average roughness (Ra) and mean square roughness (Rq) were measured by the NanoScope Analysis software v1.8 (Bruker, Germany). The sample was washed with water in an ultrasonic bath and dried at room temperature before the observation.

#### Water Contact Angle Measurements

Water contact angle (WCA) measurements were carried out using the sessile-drop method on an optical contact angle measuring device (Biolin Theta Flex, Sweden). Five droplets of 2 μl ultrapure water were dropped onto each surface and the obtained values were used to calculate means and standard deviations. The experiments were conducted in triplicate and the mean ± standard deviations of two independent experiments were calculated.

### Cell Culture

The murine-derived macrophage cell line RAW264.7 (China Center for Type Culture Collection, Shanghai, China) and human gingival fibroblasts (HGFs) (iCell Bioscience, Shanghai, China) were utilized in this study. For the macrophage experiments, RAW264.7 cells were seeded on 1) BMZ, 2) SGZ, and 3) CSGZ surfaces in 24-well plates containing Dulbecco's modified Eagle's medium (DMEM) supplemented with 10% fetal bovine serum (FBS) and 1% penicillin/streptomycin (HyClone, Thermo Fisher Scientific Inc.) at 37°C in a humidified 5% CO_2_ atmosphere. HGFs were cultured in conditioned media in a humidified atmosphere of 95% air and 5% CO_2_.

### Collection of Conditioned Media

The collection of conditioned media is used to mimic the *in vivo* microenvironment in which the macrophage on nanostructured zirconia surfaces secretes pro- and anti-inflammatory cytokines to influence the behavior of gingival fibroblasts. Briefly, RAW264.7 cells were cultured on BMZ, SGZ, and CSGZ surfaces in 24-well plates at a density of 10^5^ cells per well. After 3 days, the culture medium was collected and centrifuged at 1,500 rpm for 20 min at 4°C to remove the cell debris, and frozen at −80°C until experimental seeding. The conditioned medium is the mixture of culture medium from macrophage and DMEM at a ratio of 1:1.

### Behavior of Macrophage on Nanostructured Zirconia Surfaces

#### Adhesion and Proliferation Assay

RAW264.7 cells were seeded on TCP, BMZ, SGZ, and CSGZ surfaces in 24-well plates at a density of 2 × 10^4^ cells per well. At time points 2, 4, and 8 h after seeding, cells were rinsed with PBS to remove the unattached cells and fixed in 4% formaldehyde for 10 min followed by counterstaining with DAPI. Images were taken on a confocal laser scanning microscope (Leica TCS SP8, Germany). Ten fields of view were taken per sample at random, and nuclei were counted using the ImageJ software (Maryland, USA). The Cell Counting Kit-8 assay (CCK8, Dojindo, Kyushu, Japan) was selected to detect the proliferation of RAW264.7 at preset time points (1 and 3 days). In brief, at each time point, the medium was discarded, and the cells were rinsed with PBS. Each well was filled with 100 μl medium and 10 μl of CCK8 solution. After incubation at 37°C for 1 h, the culture medium was transferred to a 96-well plate, and the absorbance was measured using a microplate reader (Multiskan FC, Thermo Fisher) at 450 nm.

### The Polarization Gene Expression

Total RNA from RAW264.7 was isolated using TRIzol (Invitrogen) according to the manufacturer's instructions at day 3. The concentrations of RNA were quantified using NanoDrop 2000 (Thermo Fisher Scientific), then 1 μg RNA from each sample was used for reverse transcription to cDNA using OligodT and AMV reverse transcriptase (TaKaRa, Japan). Real-time RT-PCR was performed using QuantiFast SYBRGreen PCR Kit (QIAGEN, Venlo, Holland) and quantified on a CFX Connect Real-Time PCR Detection System (Bio-Rad). All samples were assayed in triplicate with three independent experiments performed. The sequences of primers for M1 macrophage polarization markers (TNF-α, iNOS, IL-1β, and CCR7), M2 macrophage polarization markers (IL-10, TGF-β, and CD206) and GAPDH used as a housekeeping gene are listed in Table 1.

**Table 1:** Primer sequences used in the qRT-PCR.

**Genes**	**Forward Primer sequences (5′-3′)**	**Reverse Primer sequences (5′-3′)**
GAPDH (M)	TGACCACAGTCCATGCCATC	GACGGACACATTGGGGGTAG
TNF-α (M)	CTGAACTTCGGGGTGATCGG	GGCTTGTCACTCGAATTTTGAGA
iNOS (M)	CAGAAGTGCAAAGTCTCAGACAT	GTCATCTTGTATTGTTGGGCT
IL-1β (M)	TGGAGAGTGTGGATCCCAAG	GGTGCTGATGTACCAGTTGG
CCR7 (M)	ATGACGTCACCTACAGCCTG	CAGCCCAAGTCCTTGAAGAG
IL-10 (M)	GAGAAGCATGGCCCAGAAATC	GAGAAATCGATGACAGCGCC
TGF-β (M)	GTGGAAATCAACGGGATCAGC	CAGCAGTTCTTCTCTGTGGAGC
CD206 (M)	AGACGAAATCCCTGCTACTG	CACCCATTCGAAGGCATTC
GAPDH (H)	CGCTGAGTACGTCGTGGAGTC	GCTGATGATCTTGAGGCTGTTGTC
COL-I (H)	GTGAACCTGGTCAAACTGGTCCTG	CCTGTGGTCCAACAACTCCTCTCT
VCL (H)	TCAGATGAGGTGACTCGGTTGG	TTATGGTTGGGATTCGCTCACA
FN (H)	AAGCCCATAGCTGAGAAGTGTTTTG	GGATGTCCTTGTGTCCTGATCGT

*M, murine; H, human*.

### Releasing Profile of Cytokines

The concentrations of TNF-α and IL-10 in the supernatants of the culture medium were determined by ELISA kits (ab100747, ab100697, Abcam, USA) following the manufacturer's instructions. The culture medium was collected at day 3 and centrifuged at 1,500 rpm for 20 min at 4°C to remove cell debris, and the supernatant was used for protein quantification.

### Behavior of HGFs Under the Conditioned Media From Macrophage Culture on Nanostructured Zirconia Surfaces

#### Morphology Observation and Proliferation Assay

HGFs were seeded at a density of 10^4^ cells per well on TCP, BMZ, SGZ, and CSGZ surfaces in 24-well plates with or without CM and cultured for 4, 8, and 24 h for morphology observation as well as for 1, 3, and 5 days for the proliferation assays. At time points 4, 8, and 24 h for cell morphology observation, cells were gently washed with PBS three times to remove the unbound gingival fibroblasts. The attached cells were fixed in 4% PFA for 15 min, treated with 0.5% Triton X-100 in PBS for 5 min, and incubated with fluorescein isothiocyanate-phalloidin (Solarbio) for 30 min. The nuclei were subsequently stained with DAPI for 10 min at room temperature. Finally, images were taken on a confocal laser scanning microscope (Leica TCS SP8, Germany). The CCK8 assay (Dojindo, Kyushu, Japan) was selected to characterize the proliferation of HGFs at preset time points (1, 3, and 5 days). In brief, at each time point, the medium was discarded, and the cells were washed with PBS. Each well was filled with 100 μl medium and 10 μl of CCK8 solution. After incubation at 37°C for 2 h, the culture medium was transferred to a 96-well plate, and the absorbance was measured using a microplate reader (Multiskan FC, Thermo Fisher) at 450 nm.

#### Apoptosis Assay

HGFs were seeded onto each sample at a concentration of 5 × 10^4^ cells per well with or without CM. After 3 days of incubation, the cells on each sample were harvested, washed twice with ice-cold PBS, resuspended in binding buffer, and stained with Annexin V-APC for 15 min in darkness and with PI for 5 min on ice. The cells were then analyzed by flow cytometry (BD Biosciences, San Jose, CA, USA). Both Annexin V+/PI– (early apoptotic) and Annexin V+/PI+ (late apoptotic) cells were included in apoptotic death determinations. The flow cytometry data were analyzed using FlowJo 10.0.7 software (USA).

### Adhesion-Related Gene Expression

HGFs were cultured onto each sample in 24-well plates at a density of 5 × 10^4^ cells per well with or without CM for real-time PCR experiments. After 3 days of culture, total RNA was isolated from HGFs for the detection of the expression of collagen-I (COL-I), fibronectin (FN), and vinculin (VCL) genes using qRT-PCR. GAPDH used as a housekeeping gene is listed in Table 1. Experiments were performed in triplicate with three independent experiments.

#### Immunofluorescence Staining Assay

The secretion and deposition of COL-I, VCL, and FN by HGFs cultured with CM or without were visualized on zirconia surface using immunofluorescence staining. All samples were fixed with 4% PFA for 15 min, permeabilized with 0.5% Triton X-100 for 5 min, and subsequently blocked with 5% bovine serum albumin (BSA) in PBS for 30 min. After blocking, the samples were incubated overnight in a primary antibody solution diluted with 5% BSA in PBS with rabbit anti-COL-I (ab34710, Abcam, USA), rabbit anti-VCL (ab129002, Abcam, USA), and rabbit anti-FN (ab2413, Abcam, USA) at 4°C. After washing twice with PBS, the samples were incubated in the dark for 1 h with the secondary antibody solution (1:200 Alexa Fluor 488/594 goat anti-rabbit IgG [H + L], Bioss). Finally, cells were reacted with DAPI for 10 min. After each step, the cells were washed with PBS three times. Ten images were taken on a confocal laser scanning microscope (Leica TCS SP8, Germany).

#### Western Blot Analysis

HGFs were cultured onto each sample in 24-well plates at a density of 5 × 10^4^ cells per well with or without CM for Western blot experiments. After 7 days of culture, HGFs were lysed in RIPA buffer and proteins were separated on 8% SDS-PAGE gels. Proteins were transferred onto polyvinylidene difluoride (PVDF) membranes which were blocked for 1 h by QuickBlock buffer (Beyotime, Jiangsu, China) at room temperature. Membranes were washed in TBST and probed overnight at 4°C with one of the following primary antibodies: collagen-I (ab34710, 1:2,000; Abcam, USA), vinculin (ab129002, 1:10,000; Abcam, USA), fibronectin (ab2413, 1:1,000; Abcam, USA), and β-actin (1:1,000, ab8227; Abcam, USA). Membranes were washed in TBST and incubated for 1 h with HRP-conjugated secondary antibodies at room temperature. Proteins were visualized using the ECL system (Beyotime, Jiangsu, China).

### Statistical Analysis

All statistical calculations were performed with GraphPad software v.6 (GraphPad Software, La Jolla, CA, USA). All data are expressed as mean ± standard deviation (SD). Differences between groups were analyzed using analysis of variance (ANOVA) followed by Bonferroni test. A *p* < 0.05 was considered statistically significant.

## Results

### Surface Characteristics

**Figure 1:**
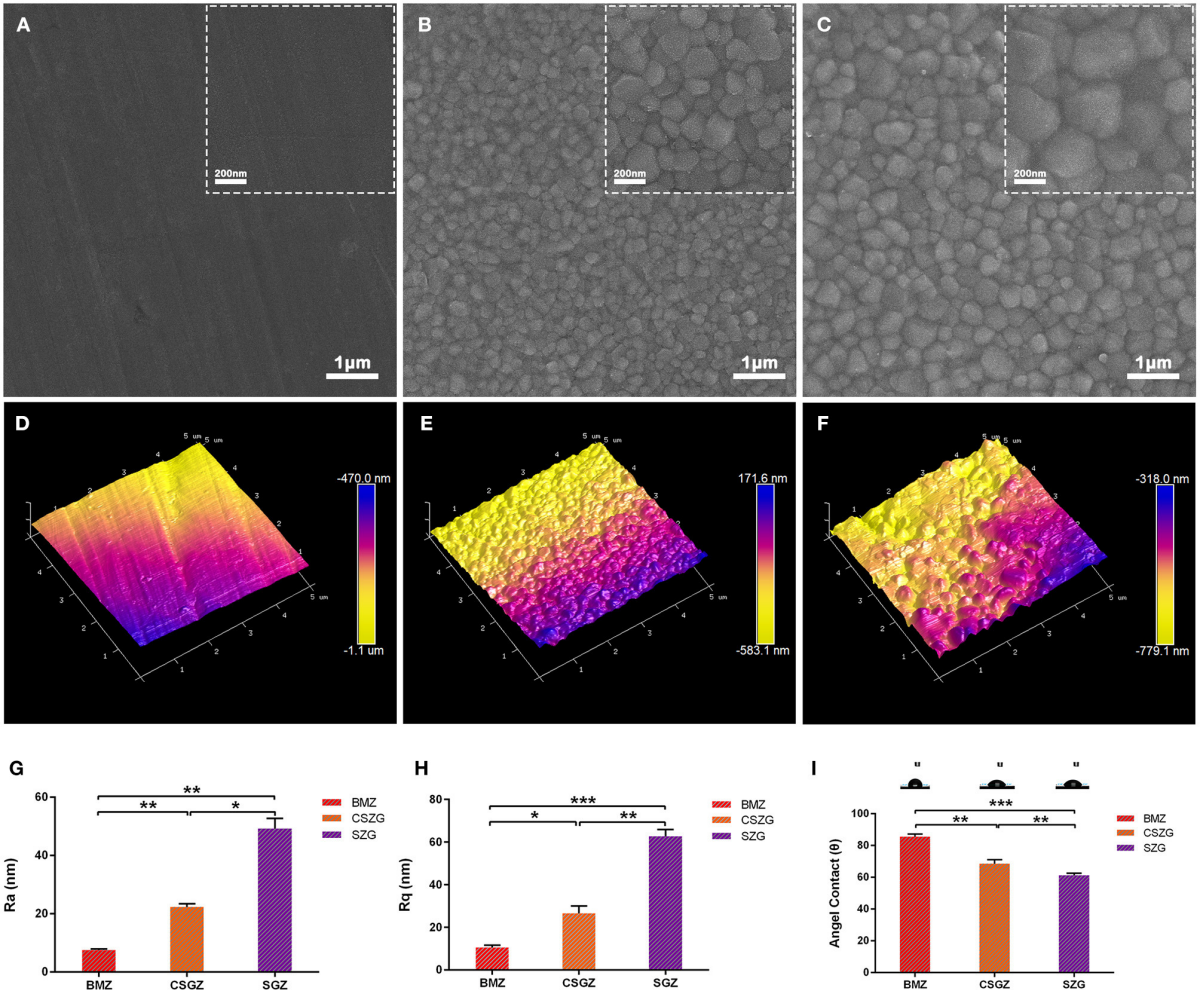
Surface measurements and analysis of nanostructured zirconia surfaces. **(A–C)** Representative SEM images show the morphology of nanostructured surfaces (original magnifications are ×50,000 for larger- and ×20,000 for smaller-scale bar images). **(D–F)** Representative AFM images show the topography of nanostructured surfaces. **(G,H)** Roughness of nanostructured zirconia surfaces measured by AFM. **(I)** Hydrophilicity of nanostructured zirconia surfaces analyzed by water contact angle. Data are means ± SE. *p < 0.05, **p < 0.01, ***p < 0.001.

Surface morphology of nanostructured zirconia surfaces was observed by using SEM (Figures 1A–C). Blank-machined zirconia surfaces showed flat surfaces with regular nanogrooves, while self-glazed zirconia and coated self-glazed zirconia surfaces revealed a shaggy and irregular structure with random distribution of coarse zirconia crystalline owing to a 3D local plastic deformation process. CSGZ surfaces appeared to display an ultrafine grain and become smoother than SGZ after the coating procedure. The atomic force microscopy (AFM) data shown in Figures 1D–F were in good agreement with the Ra values. BMZ presented the lowest surface roughness among the samples tested (Ra: 7.6 ± 0.3 nm; Rq: 10.6 ± 1.1 nm), which was statistically significantly different (*p* < 0.01) compared with CSGZ surfaces (Ra: 22.0 ± 1.1 nm; Rq: 26.7 ± 3.4 nm), as well as with SGZ (Ra: 49.3 ± 3.5 nm; Rq: 62.7 ± 3.3 nm). Moreover, SGZ surfaces presented statistically significantly higher roughness compared with CSGZ surfaces (*p* < 0.05 for parameters Ra and Rq) (Figures 1G,H). The wettability of nanostructured zirconia surfaces was determined by water contact angle measurements. The mean angles of BMZ and CSZG surfaces were 85.55 and 68.67°, respectively, while SZG surfaces showed favorable hydrophilicity with a relatively lower water contact angle of ~61.19° after coating (Figure 1I).

### Macrophage Adhesion and Proliferation

**Figure 2:**
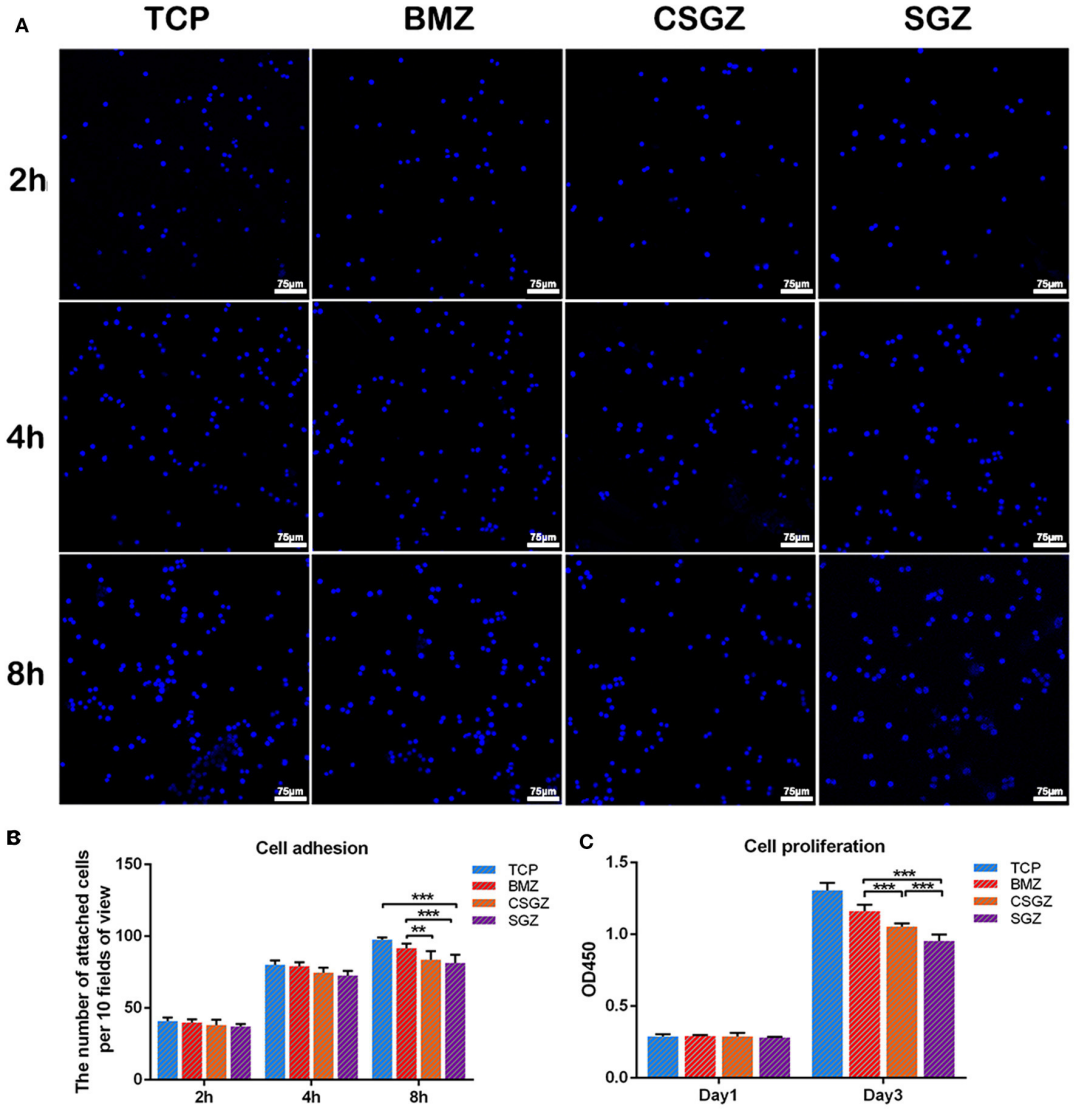
Effect of nanostructured zirconia surfaces on RAW264.7 cell adhesion and proliferation for (1) tissue culture plastic (TCP), (2) BMZ, (3) CSGZ, and (4) SGZ samples. **(A,B)** Cell adhesion images and the number of attached cells at 2, 4, and 8 h. **(C)** Cell proliferation at 1 and 3 days. Data are means ± SE. **p < 0.01, ***p < 0.001.

The effect of nanostructured zirconia surface was investigated on macrophage adhesion and proliferation (Figure 2). It was observed that all surface topographies demonstrated high attachment, and no significant differences were observed among all groups at 2 or 4 h post-seeding. At time point 8 h, cell attachment on BMZ surfaces showed significant differences from CSGZ and SGZ surfaces (Figures 2A,B). Analysis of cell proliferation demonstrated that no differences were observed among all the groups at day 1 post-seeding. At 3 days, however, macrophages seeded on BMZ and CSGZ surfaces showed significantly higher cell proliferation when compared with SGZ surfaces (*p* < 0.001; Figure 2C). TCP demonstrated significantly the highest cell proliferation at 3 days when compared with all the other groups (*p* < 0.001; Figure 2C).

### Macrophage Polarization Gene Expression

**Figure 3:**
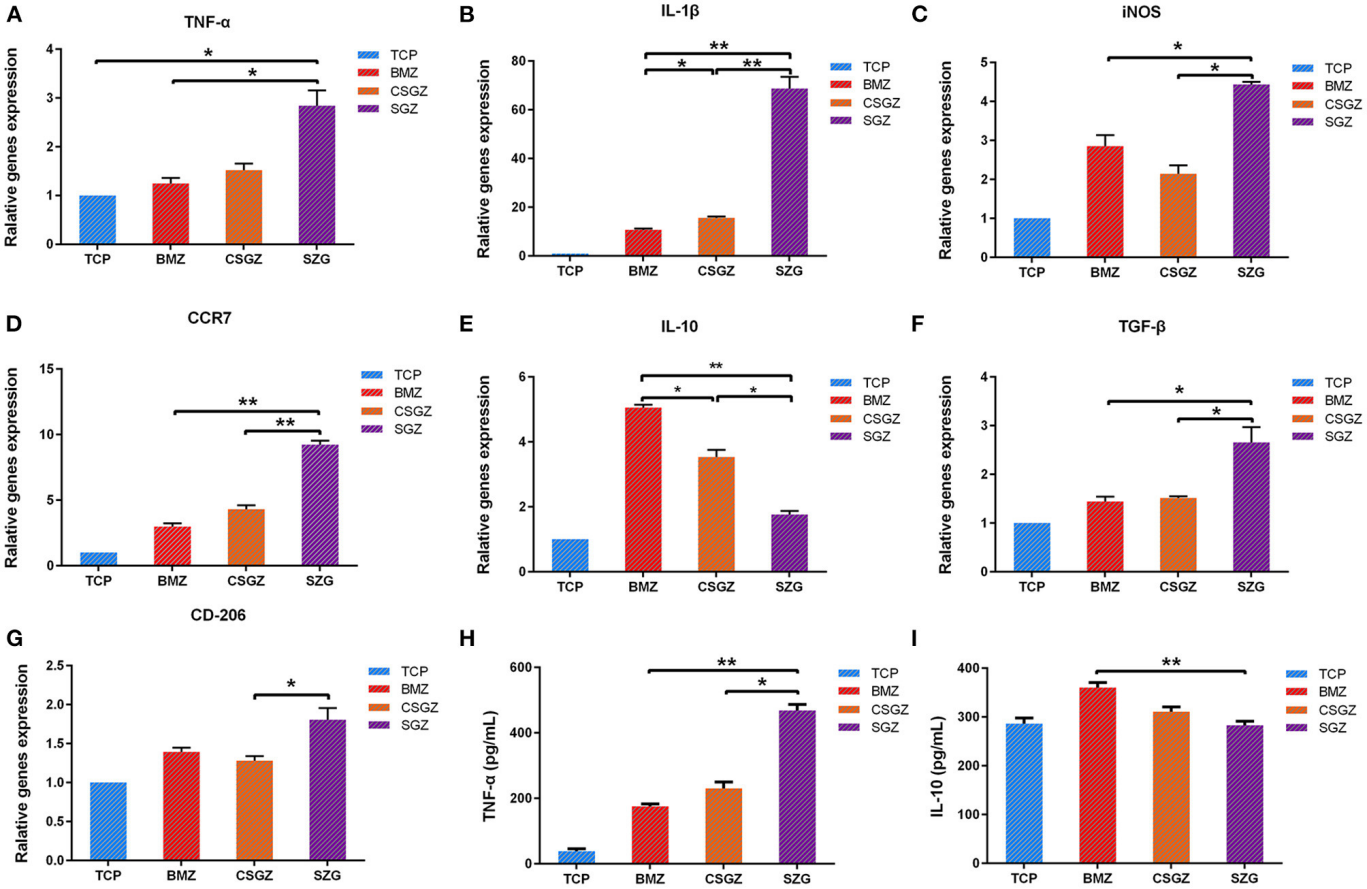
RAW264.7 cell polarization gene expression. **(A–G)** The expression of related polarization genes: TNF-α, IL-1β, iNOS, CCR7, IL-10, TGF-β, and CD206 in RAW264.7 cultured on nanostructured zirconia surfaces at 3 days. GAPDH was used as housekeeping genes. **(H,I)** Quantified TNF-α and IL-10 secretion from macrophages cultured on different nanostructured zirconia surfaces at 3 days using ELISA assay. Data are means ± SE. *p < 0.05, **p < 0.01.

The expression levels of polarization genes produced by macrophage cells are believed to modulate soft tissue healing. The expression of IL-1β, iNOS, and CCR7 was markedly higher in the macrophage cultured on the SGZ surfaces compared with the TCP, BMZ, and CSGZ surfaces at 3 days post-seeding (*p* < 0.05; Figures 3B–D). The expression of TNF-α was higher on SGZ surfaces compared with BMZ surfaces (*p* < 0.05; Figure 3A). The expression levels of M2 macrophage polarization genes (IL-10, TGF-β, and CD206) are shown in Figures 3E–G. There was a significant difference in the TGF-β expression level on SGZ surfaces compared with BMZ and CSGZ surfaces. The expression level of CD206 on SGZ surfaces showed statistical difference compared with CSGZ surfaces (*p* < 0.05; Figure 3G). Conversely, the cells showed the strongest expression of IL-10 on the BMZ surfaces with statistical differences among samples at 3 days of culture (*p* < 0.05; Figure 3E).

### Pro- and Anti-inflammatory Cytokine Production

RAW264.7 cells grown on the SGZ surfaces secreted significantly greater TNF-α into cell culture media at 3 days than the BMZ and CGGZ surfaces (*p* < 0.05; Figure 3H). There were no differences in the production of anti-inflammatory (IL-10) cytokines on the nanostructured zirconia surfaces (TCP, CSGZ, and SGZ) at 3 days. Interestingly, BMZ surfaces markedly increased IL-10 production level in RAW264.7 cells compared with CSGZ and SGZ with rough nanostructure surfaces (*p* < 0.05; Figure 3I).

### Morphology Observation and Proliferation Assay of HGFs

**Figure 4:**
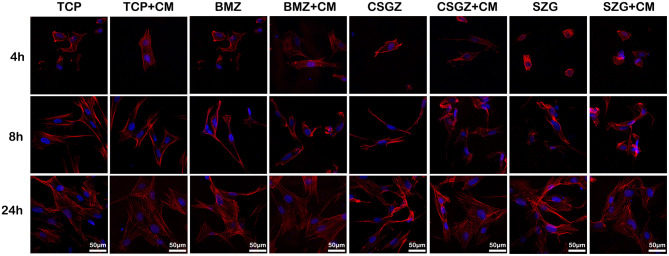
Spreading of human gingival fibroblasts cultured with or without CM on nanostructured zirconia surfaces after 4, 8, and 24 h of seeding. Cells were stained with phalloidin (red) and DAPI (blue).

**Figure 5:**
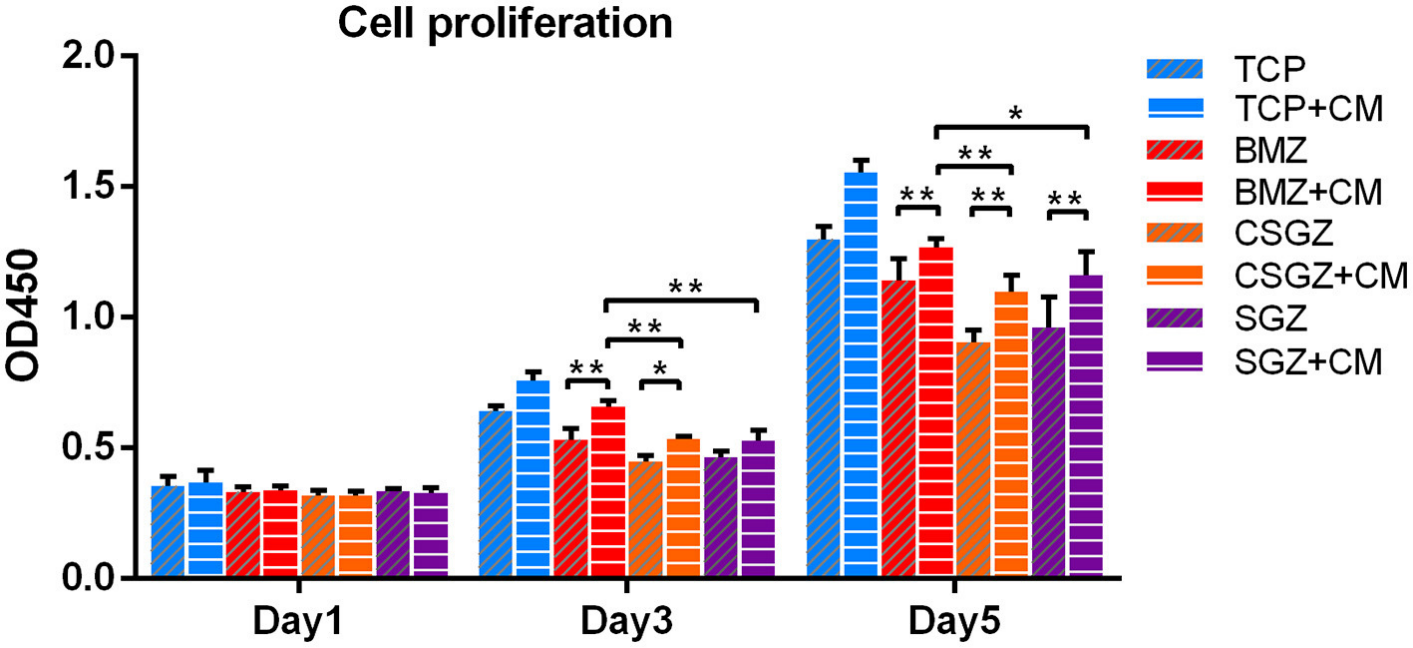
Effects of nanostructured zirconia surfaces on human gingival fibroblast proliferation cultured with or without CM at 1, 3, and 5 days. Data are means ± SE. *p < 0.05, **p < 0.01.

Thereafter, a series of experiments investigating the nanostructured surfaces with or without CM from RAW264.7 culture on HGFs behavior were performed. Figure 4 shows the morphology of HGFs cultured with or without CM at 4, 8, and 24 h. It was found that after 4 and 8 h, cells had spread on all surfaces except the SGZ surfaces with a round appearance, whereas by 24 h, cell spreading on all three zirconia surfaces became apparent. Interestingly, the addition of CM tended to promote the spreading of HGFs, especially on TCP and BMZ surfaces (Figure 4). There were no significant differences observed in cell proliferation among the samples at 1 day, while it was found that HGFs cultured with CM on CSGZ and SGZ surfaces demonstrated significantly lower cell proliferation in comparison with BMZ surfaces at both 3 and 5 days (*p* < 0.05; Figure 5). However, when CM was added to the culture media, significantly higher cell proliferation was observed at 5 days on all three zirconia surfaces compared with their respective controls (*p* < 0.05; Figure 5).

### Apoptosis Assay of HGFs

**Figure 6:**
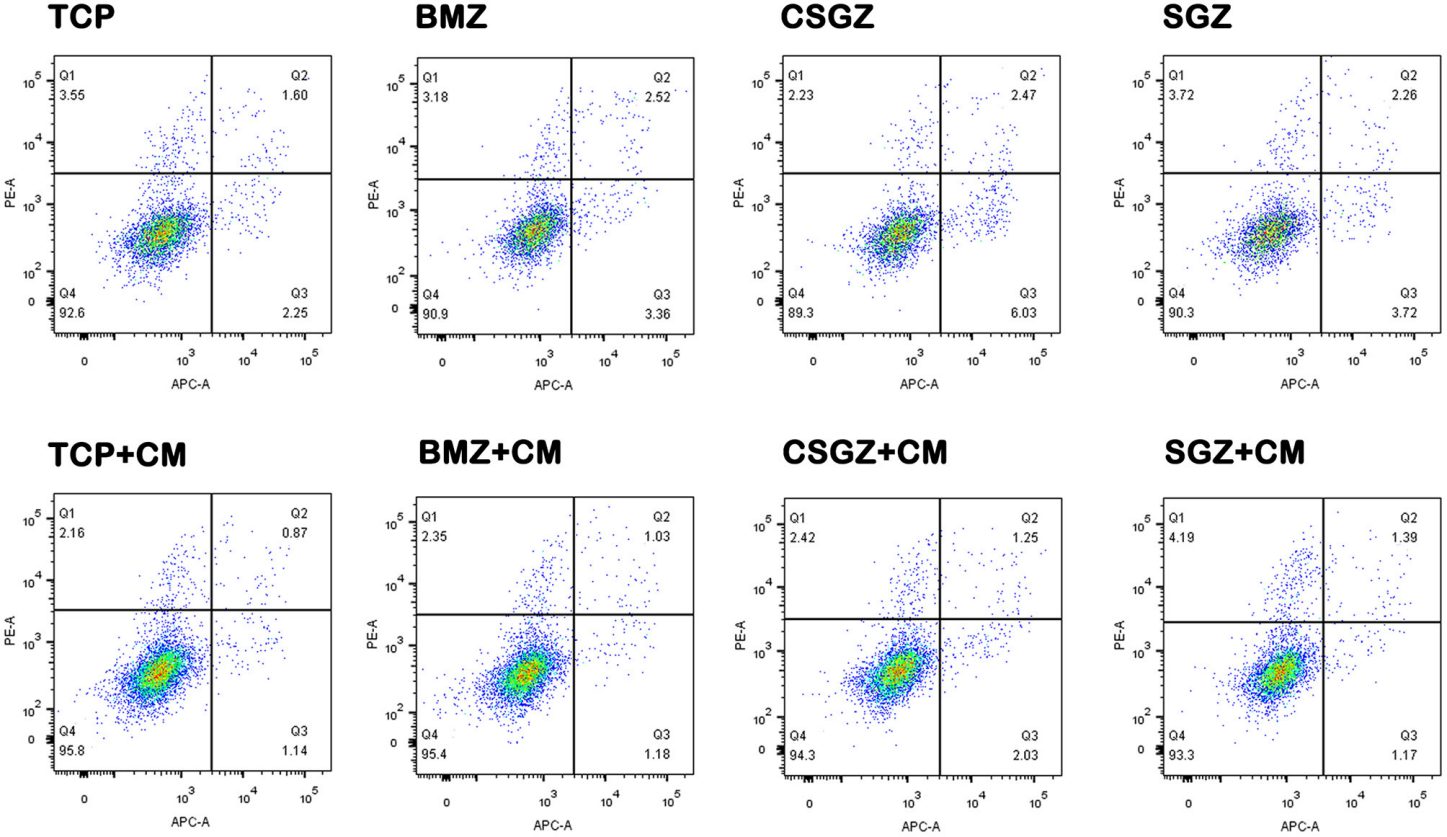
Effects of nanostructured zirconia surfaces on the apoptosis of human gingival fibroblasts cultured with or without CM. Flow cytometric analysis of human gingival fibroblast cell apoptosis after 3 days of culture with or without conditioned media on nanostructured zirconia surfaces.

The apoptotic behavior of HGFs after culturing on each sample with or without CM for 3 days was determined through a flow cytometric analysis. As shown in Figure 6, the apoptotic rates of HGFs cultured without CM on TCP, BMZ, CSGZ, and SGZ surfaces were 3.85, 5.88, 8.50, and 5.98%, respectively, while the apoptotic rates of HGFs cultured with CM were 2.01, 2.21, 3.28, and 2.56% (Figure 6), which indicate that CM reduced cell apoptosis of HGFs cultured on all sample surfaces.

### The Adhesion Gene Expression in HGFs

**Figure 7:**
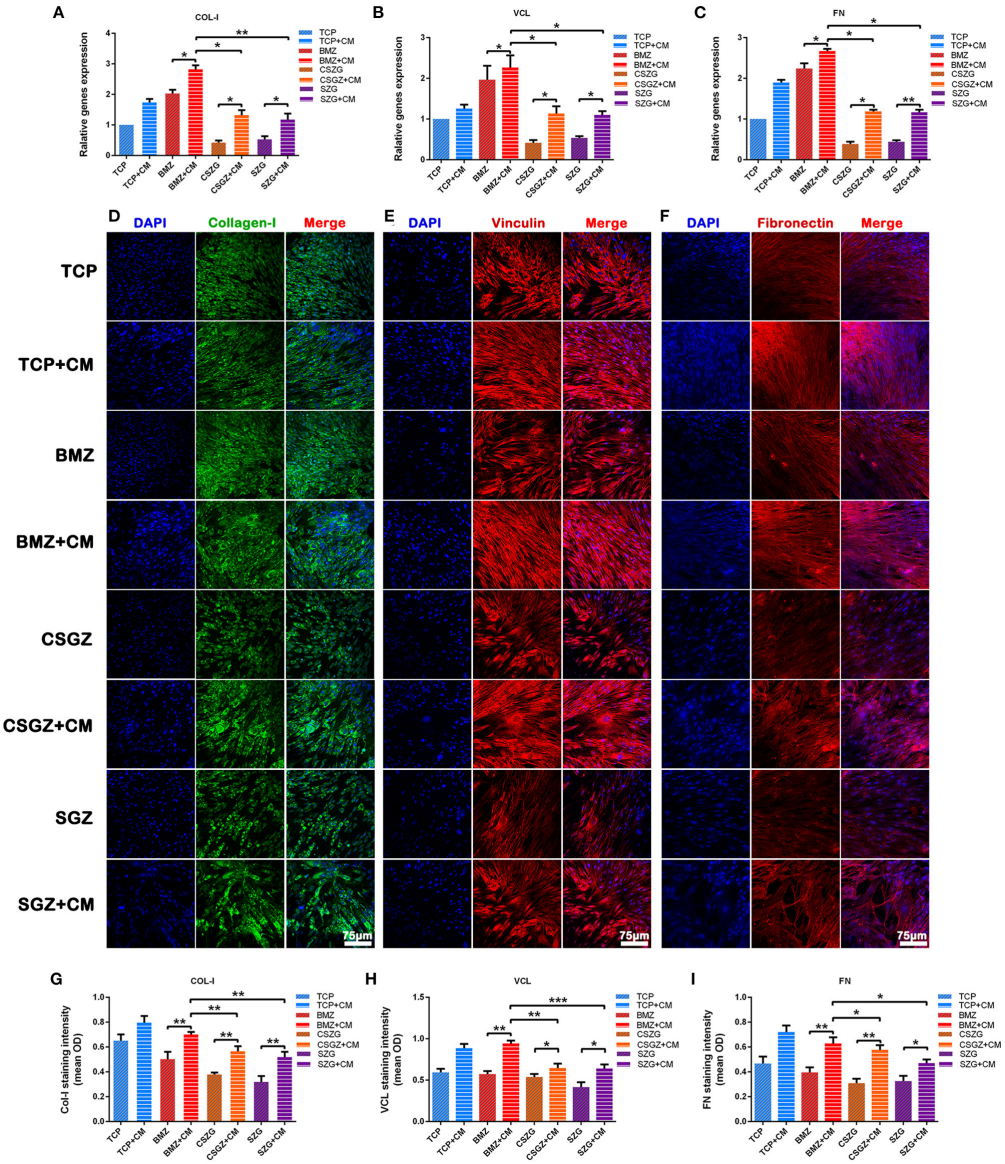
Relative gene expression and immunofluorescence staining of collagen-I, vinculin, and fibronectin. **(A–C)** The expression of adhesion-related genes: COL-I, VCL, and FN in HGFs cultured on nanostructured zirconia surfaces at 3 days. **(D–F)** Representative immunofluorescence staining images of COL-I, VCL, and FN in HGFs cultured with or without CM on nanostructured zirconia surfaces at 7 days. **(G–I)** The protein expression of COL-I, VCL, and FN by semi-quantitative analysis. Data are means ± SE. COL-I, collagen type I; VCL, vinculin; FN, fibronectin; HGFs, human gingival fibroblasts. Data are means ± SE. *p < 0.05, **p < 0.01, ***p < 0.001.

After incubation of the HGFs with or without CM for 3 days, it was observed that CM significantly upregulated the expression levels of COL-I, VCL, and FN on all sample surfaces when compared with their respective controls (*p* < 0.05; Figures 7A–C). Once again, the relative expression levels of COL-I, VCL, and FN on CSGZ and SGZ surfaces were similar and showed no significance, while BMZ surfaces had the most pronounced and detrimental effect, whereby the addition of CM significantly increased COL-I, VCL, and FN expression compared with CSGZ and SGZ surfaces (*p* < 0.05; Figures 7A–C).

### Immunofluorescence Staining Assay for the Protein Expression of HGFs

The secretion of target proteins by HGFs was confirmed semi-quantitatively by immunofluorescence staining after culturing for 7 days. The immunofluorescence staining images of COL-I, VCL, and FN were then utilized to visualize the extracellular matrix deposition of human gingival fibroblasts onto nanostructured zirconia surfaces (Figures 7D–F). The results of the semi-quantitative analysis are shown in Figures 7G–I. No differences could be observed between CSGZ and SGZ surfaces with respect to the staining intensity of COL-I, VCL, and FN. In addition, the protein distributions of COL-I, VCL, and FN on BMZ surfaces with CM appeared to be significantly higher when compared with those on CSGZ and SGZ surfaces. Furthermore, it was found that CM significantly upregulated the expression levels of COL-I, VCL, and FN on BMZ, CSGZ, and SGZ surfaces when compared with their respective controls (*p* < 0.05; Figures 7G–I).

### Western Blot Analysis for the Expression of COL-I, VCL, and FN in HGFs

**Figure 8:**
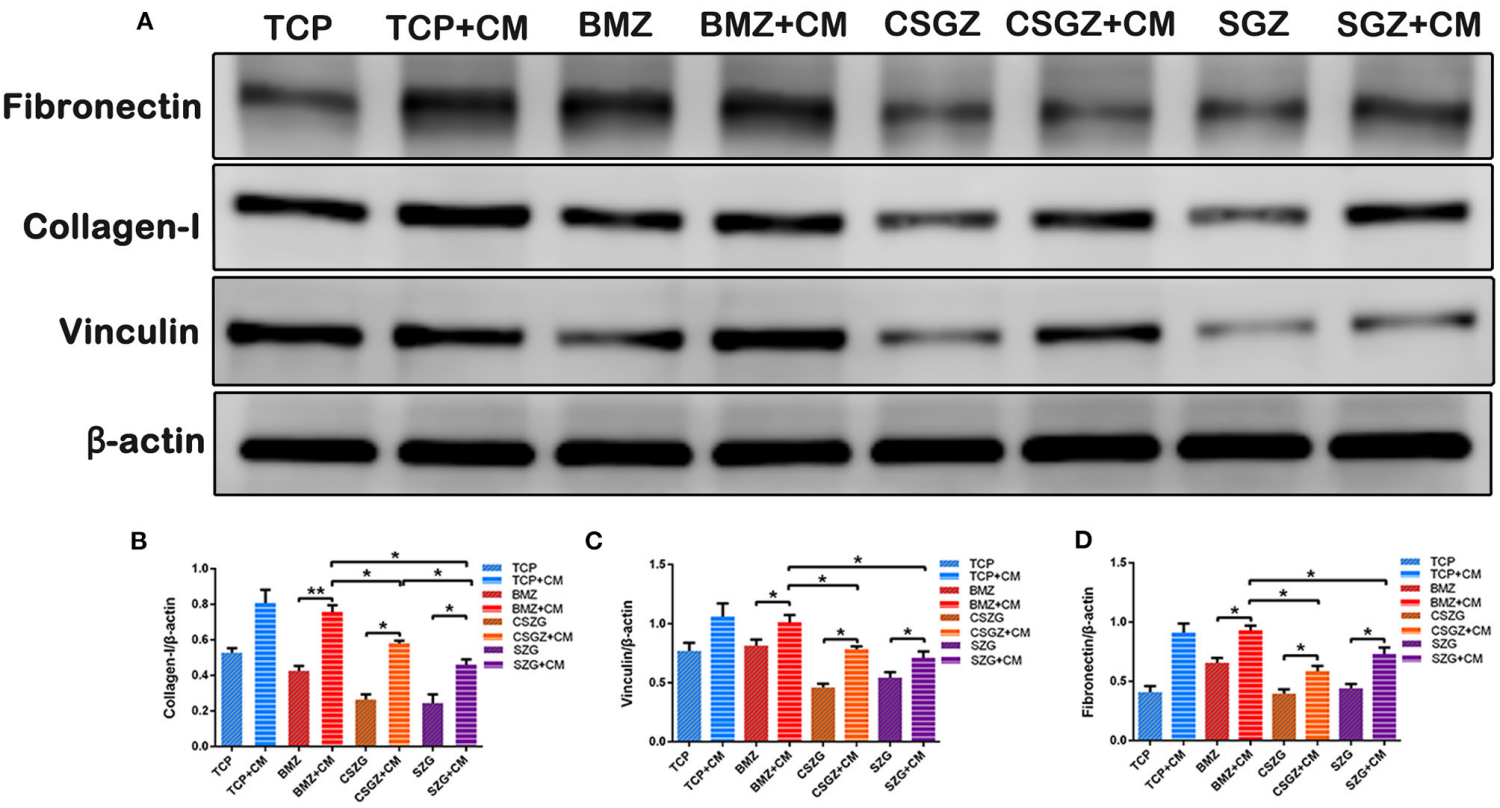
Expression of adhesion-related proteins detected by Western blot. **(A)** Representative images of COL-I, VCL, and FN expression. **(B–D)** Quantitative analysis of COL-I, VCL, and FN expression in HGFs with or without CM on nanostructured zirconia surfaces at 7 days. β-Actin was used to normalize the data. COL-I, collagen type I; VCL, vinculin; FN, fibronectin; HGFs, human gingival fibroblasts. Data are means ± SE. *p < 0.05, **p < 0.01.

The Western blotting results also demonstrated that CM notably upregulated the protein expression levels of COL-I, VCL, and FN on BMZ, CSGZ, and SGZ surfaces when compared with their respective controls, which was in accordance with immunofluorescence staining observations. Meanwhile, compared with CSGZ and SGZ surfaces, HGFs cultured with CM on BMZ surfaces expressed higher COL-I, VCL, and FN (*p* < 0.05; Figures 8A–D). There were no statistically significant differences in the VCL and FN expression between the CSGZ and SGZ surfaces (Figures 8C,D). However, the protein of COL-I on CSGZ surfaces with CM appeared to be significantly higher when compared with SGZ surfaces (*p* < 0.05; Figure 8B).

## Discussion

There is an increasing interest in the biomaterials community about the effect of macrophages on the integration around the peri-implant tissue in recent years (Brown et al., 2012). While early studies on dental implants have emphasized the necessity for the integration of soft tissues, it is of great importance to demonstrate that macrophages, one of the main immune cell types that interact with foreign implanted biomaterials, are responsible for regulating tissue remodeling including tissue integration of various biomaterials (Miron and Bosshardt, 2016). Notably, despite the many studies examining the interaction between macrophage and biomaterials in the medical field (Jia et al., 2019), their function in implant dentistry has not been completely understood. A recent systematic review investigating relevant cellular researches regarding implant surfaces discovered that there are about 90% of all published literature paying attention to mesenchymal cell behavior on implant surfaces and approximately 10% concentrated on immune cells including monocytes, macrophages, osteoclasts, leukocytes, and multinucleated giant cells (Thalji and Cooper, 2014). This finding indicates the lack of study with immune modulation of implant surfaces despite the fact that tissue integration is routinely found preceded by macrophage accumulation (Chehroudi et al., 2009).

Since macrophages arrive at the implant surfaces earlier than HGFs and can affect the HGFs' behavior, studies have been done to modify the implant surfaces by regulating macrophages to achieve better implant integration. Previous studies have revealed that alteration of the implants' surface roughness and topography can modulate macrophage functions including cell adhesion and cytokine secretion (Yim and Leong, 2005; Ainslie et al., 2009). In this study, macrophages were prone to favor adhesion on BMZ and CSGZ surfaces. It was also noteworthy that SGZ surfaces displayed less macrophage cell attachment, which is consistent with the result at later time points following macrophage proliferation (Figures 2A–C). These results are in accordance with the recent investigations which have found that a smooth surface allows macrophages to attach and spread more than on a rough surface (Hotchkiss et al., 2016). In addition, a rough surface would induce more M1 macrophages compared with a smooth surface, and the inflammatory cytokines secreted later might hinder the tissue healing process (Alfarsi et al., 2014; Hotchkiss et al., 2016). Generally, the polarization of macrophages could be induced by the addition of specific cytokines and the stimuli of the local microenvironment (Yao et al., 2019). However, there were no foreign cytokines and stimuli introduced in this study, indicating that the nanostructured surface topography could directly modulate macrophage polarization. Our results prove that macrophages on SGZ surfaces tended to induce higher M1 polarization and a lower M2 polarization was observed with a low expression of IL-10 (Figures 3A–E). By contrast, on the BMZ and CSGZ surfaces, M2 polarization was significantly higher and M1 polarization was lower than that on the SGZ surfaces on day 3. Consistently, ELISA test for TNF-α and IL-10 expressions showed that nanostructured surfaces can modulate macrophage polarization *in vitro*. At 3 days after culture, the BMZ surfaces induced both IL-10 production and TNF-α synthesis. In contrast, more TNF-α expression and less IL-10 expression cells were detected in the supernatant from the SGZ surfaces compared with those on BMZ surfaces (Figures 3H,I), which may reflect the progression of more serious inflammation on rough surfaces. In fact, the large ratio of M1 macrophages to M2 macrophages is highly related to the failure of artificial joints (Rao et al., 2012).

Interestingly, although surface roughness tends to promote a pro-inflammatory response, its effect on HGF cell behavior is controversial. Whereas fibroblasts initially are inclined to adhere better on a smooth surface, they showed rapid cell proliferation on a rough surface (Rompen et al., 2006). However, reports have shown that an implant surface with grooves can promote cell stretching and guide the cells to be aligned in parallel within the surface grooves (Mustafa et al., 2005; Pae et al., 2009). In fact, cells on a smoother surface have to stretch themselves and form a strong cytoskeletal structure so as to stabilize themselves mechanically on the topography of the surface, when compared with a rough surface (Kunzler et al., 2007). Hence, a smoother surface may facilitate more cell proliferation to the topographical “limit” of the surface. It is acknowledged that immunologic response is another factor, which could be regulated by both macrophage and surface topography (De Marco et al., 2017).

Surface topography generates an impact on macrophage polarization as well as on fibroblast behavior. Herein, we further investigated that macrophage phenotypes modulated by nanostructured zirconia surfaces could affect the interaction between the zirconia surfaces and the HGFs in a co-culture system. Our results illustrated that CM from RAW264.7 culture on BMZ, CSGZ, and SGZ surfaces more strongly enhanced the proliferation of HGFs as compared with their respective controls at 5 days (Figure 5). Additionally, CM from RAW264.7 culture on BMZ, CSGZ, and SGZ surfaces upregulated adhesion gene expression and secretion of COL-I, VCL, and FN compared with their respective controls (Figures 7A–I). Meanwhile, HGFs cultured under collected CM on BMZ surfaces tend to display a higher expression and secretion of COL-I, VCL, and FN than those on CSGZ and SGZ surfaces (Figures 7A–I). These results could explain the favorable modulation effects of macrophages cultured on BMZ surfaces on tissue integration. In response to different implantable biomaterials, macrophages with different phenotypes vary the production of cytokines, chemokines, growth factors, and other molecules that contribute to the local milieu and further modulate tissue activities to regulate the function of target cells (Jia et al., 2019). Once activated, macrophages secrete various bioactive components, including growth factors, cytokines, and exosomes, according to their specific phenotypes (Das et al., 2015). After taking the conditioned medium into consideration, the apoptotic rate of fibroblasts on TCP, BMZ, CSGZ, and SGZ surfaces reduced by 1.84, 3.67, 5.22, and 3.42%, respectively, compared with their respective controls (Figure 6), which indicated that CM collected from RAW264.7 culture in all samples slightly inhibited the apoptotic effect on fibroblasts. The conditioned medium from M2 macrophages has been shown to affect fibroblast behavior which secrete extracellular matrix (ECM), specifically collagen that can enhance efficacious implant integration (Fujioka-Kobayashi et al., 2020). Our Western blotting results showed that fibroblasts cultured with CM on BMZ surfaces produce more ECM with a higher expression of COL-I, VCL, and FN as compared with those on CSGZ and SGZ surfaces (Figures 8A–D). In addition, the CM upregulated the gene expression levels of COL-I, VCL, and FN on BMZ, CSGZ, and SGZ surfaces (Figures 7A–C), when compared with their respective controls, which was in accordance with the immunofluorescence staining results (Figures 7D–I). Therefore, macrophages may play a prominent role in tissue integration. The strategies adopted to better regulate macrophage immune response through altering the surface topography of biomaterials are essential and necessary.

Surface wettability is another vital factor that influences cell adhesion on the biomaterials' surface (Rupp et al., 2004). Indeed, a more hydrophilic surface is usually accompanied by a favorable wettability on the basis of the Wenzel law, but it has been demonstrated that wettability did not increase in accordance with roughness in some situations (MacDonald et al., 2002). In our case as shown in Figure 1I, the initial contact angle value of SGZ was 61.19, and after decreasing the grain size, a more hydrophobic surface was observed with an angle of 68.67° in CSGZ. The result of this study is in accordance with that of previous studies, which found that wettability decreased with the decrease in grain particle size (Karunakaran et al., 2015; Youshia et al., 2017). According to the literature, the wettability of the material surface will control the proteins' ability to adsorb onto the surface and there will be a formation of blood clot and fibrin network (Kopf et al., 2015). A related research shows that an increase in anti-inflammatory cell response has been shown to occur with increased wettability of the surface (Zhou et al., 2015). In particular, it was found that a switch of macrophage phenotype from M1 toward M2 at the implant–tissue interface by means of surface modifications is instrumental for wound healing and tissue integration (Ma et al., 2014). Therefore, changing surface wettability may act as an applicable approach to control macrophage phenotype and further improve tissue healing.

During implantation, an early-stage immunological response begins with protein adsorption on the extraneous implant surface, which can subsequently modulate macrophage recognition and activation and, eventually, evoke an adverse foreign body reaction (FBR) (Mariani et al., 2019). Macrophages fuse to form foreign body giant cells (FBGC), which is a crucial feature of FBR in case that they fail to internalize foreign biomaterial through phagocytosis (Trindade et al., 2016). Since a dense fibrous capsule forming during the process of FBR could obstruct the oxygen and nutrient exchange between the host and the biomaterial, causing the biomaterial to be non-functional, effective strategies to modify or optimize FBR are of fundamental importance for the advancement of implant materials (Zhou et al., 2015). The results obtained in the present study further emphasize that macrophages have a crucial regulator effect on cell function and behavior. To this end, future researches should be performed to elucidate the pivotal interaction between the host immune response and the biomaterial.

## Conclusions

In this study, we discovered that BMZ surfaces were superior to macrophage proliferation and adhesion. All nanostructured zirconia surfaces induced macrophage polarization toward both inflammatory M1 and anti-inflammatory M2 phenotype with more M2 macrophage phenotype on BMZ surfaces. Meanwhile, our studies confirmed that CM from RAW264.7 culture on three nanostructured zirconia surfaces upregulated HGFs' functions including cell proliferation and ECM formation, suggesting enhanced soft tissue integration abilities of immunoregulation. Collectively, the synergistic regulation of surface topography and CM could enhance HGFs' behavior with higher expression of COL-I, VCL, and FN on BMZ surfaces. After coated with a nano zirconia film, CSGZ surfaces showed some difference in cell proliferation, adhesion, and protein production compared with SGZ surfaces. Our present study suggests that a favorable immune microenvironment can be developed by macrophage modulation through the functional surface design of biomaterials and, subsequently, regulate the behavior and function of progenitor cells.

## Data Availability Statement

The original contributions presented in the study are included in the article/supplementary materials, further inquiries can be directed to the corresponding author/s.

## Author Contributions

ZW contributed to the conception and design of the study. JW performed the experiments. PY and HL carried out the data analysis. All authors participated in the drafting of the manuscript and critical revision of the draft. All authors have read and approved the final version of the manuscript.

## Conflict of Interest

The authors declare that the research was conducted in the absence of any commercial or financial relationships that could be construed as a potential conflict of interest.
